# Stressful Events and Adolescent Psychopathology: A Person-Centred Approach to Expanding Adverse Childhood Experience Categories

**DOI:** 10.1007/s40653-021-00392-8

**Published:** 2021-07-30

**Authors:** Justin MacLochlainn, John Mallett, Karen Kirby, Paula McFadden

**Affiliations:** 1 School of Psychology, Ulster University, Room H259, Cromore Road, Coleraine campus, Co. Derry, BT52 1SA Coleraine, Northern Ireland; 2 School of Psychology, Ulster University, Room H245, Cromore Road, Coleraine campus, Co. Derry, BT52 1SA Coleraine, Northern Ireland; 3 School of Applied Social and Policy Sc, Institute for Research in Social Sciences, Magee campus, Room MF211, Derry, BT48 7JL Northern Ireland

**Keywords:** Stress, Adolescent, Psychopathology, Adverse childhood experience, Anxiety, Depression, Dose–response, Latent Class

## Abstract

Stress from cumulative adverse childhood experiences (ACEs) can pose a serious risk of experiencing anxiety, depression, and other mood disorders in adolescence. However, there is a paucity of research identifying specific profiles or combinations of exposure to other forms of stressful life events and their impact on adolescent psychopathology. This study attempted a conceptual expansion of the ACE checklist by examining these stressful events. The study used cross-sectional data from a modified version of the CASE Study survey where 864 adolescents (56% female, n = 480), aged from 11 – 18 years were recruited from four post-primary schools in the North-West region of NI. Latent class analysis of the 20-item stressful events checklist revealed 3 distinct risk classes: a low-risk class (53.5%), at-risk class (42.7%), and an immediate-risk class (3.8%). Results showed those at most risk of adolescent psychopathology had the highest probability of encountering interpersonal relationship issues, experiencing family dysfunction, and having close friends experiencing psychological difficulties. Findings indicate that the original ten ACE categories may be too narrow in focus and do not capture the wide range of childhood adversity. Expanding the ACE checklist to include other stressful events is discussed as these may also be antecedents to psychopathologic responses.

## Introduction

Stressful events are commonly defined as occurring when the demands of any given situation threaten to surpass the resources held by the individual (Lazarus & Folkman, 1984). Childhood adversity denotes a wide range of stressful and traumatic events, These events may pose a serious risk to the young persons’ physical and psychological well-being (Cook et al., 2017; Petruccelli et al., 2019), and are associated with increased risk of both internalising and externalising problems at multiple time points across the life-span (Chapman et al., 2007; Little & Akin-Little, 2013), higher comorbidity (McChesney et al., 2015), and recurrence of psychopathology (Benjet et al., 2010; Clark et al., 2010).

Findings from the original Adverse Childhood Experiences (ACE’s) study (Felitti et al., 1998) has led to a surge of studies replicating and advancing evidence of ways that stress from cumulative childhood adversities can severely impact and diminish quality of life (Bellis et al., 2014a; McGavock & Spratt, 2012; Ramiro et al., 2010; Schilling et al., 2007). ACEs are defined as a traumatic or stressful event that an individual has experienced before their eighteenth birthday. These events include physical, emotional, and sexual abuse, neglect, domestic violence, substance abuse, mental illness, parental separation, and incarceration (Felitti et al., 1998). Much of the ACE literature has focused on cumulative risk and presented an overall ACE score and its subsequent negative impact on health outcomes in adulthood. For example, those with four or more ACEs reported three times the rate of heart disease, almost five times the rate of depression, and twelve times the rate of suicide in comparison to those with no ACEs (Bellis et al., 2014b; Felitti et al., 1998).

More recently, studies have begun to illustrate how individual ACE categories, including adverse social environment categories, such as poverty or poor housing, which were absent from the original ACE checklist, independently predict concurrent health outcomes including poorer emotional and behavioural functioning, and lower cognitive functioning in adolescence (Ballard et al., 2015; Coley et al., 2013). Indeed, Marryat and Frank (2019), using data from the ‘Growing up in Scotland’ birth cohort study demonstrated that ACEs were highly correlated with socioeconomic disadvantage (Marryat & Frank, 2019). Accumulating evidence of the deleterious effects of a single independent adversity category on adolescent psychopathology is important as it informs targeted screening, prevention, and intervention for individuals and their communities (Lanier et al., 2018). Likewise, studies reporting dose–response relationships between cumulative ACE scores and mental health outcomes are adding to the volume of mounting evidence on the relationship between accumulated stress and psychopathology (Chapman et al., 2004; Dube et al., 2001).

However, a limitation of the ACE score is the assumption that categories of adversity are of equal weight (Anda et al., 2020). Additionally, attempts to understand the impact of stress from single adversity categories may prove problematic (Shevlin & Elklit, 2008) with contemporary research indicating childhood adversities often co-occur (Armour et al., 2014; Creamer et al., 2001). Therefore, classifying specific sub-types of adversity exposure, and subsequent mental health outcomes associated with these sub-types can make a valuable contribution to the clinical and theoretical evidence base in order to inform and transform services for trauma-impacted youth (Barboza, 2018; Lanier et al., 2018; McGavock & Spratt, 2012).

Using latent class analysis (LCA), researchers have been attempting to show different profiles or combinations of adversity suggesting different pathways to outcomes depending on the types and combinations of childhood adversities experienced (Lanier et al., 2018; McChesney et al., 2015). LCA is a statistical method used to categorize underlying relationships or sub-types between observed variables (Shevlin & Elklit, 2008). It is a person-centered approach that identifies individual responses to each variable and identifies unobserved sub-classes of individuals depending on observed item endorsement (Wang & Wang, 2019). This method has been used to classify ACE endorsement in several recent studies that found childhood adversities associated with maladaptive family functioning (e.g., parental mental illness, child abuse, neglect) was the strongest predictor of the onset and persistence of mental health problems (McLaughlin et al., 2010; Kessler et al., 2010). Others have shown that membership of a poly-adversity or high ACEs class led to greater psychopathologic response from respondents (Barnes et al., 2009; Lew & Xian, 2019; McLafferty et al., 2015).

However, there is a paucity of research identifying specific profiles or combinations of exposure to other stressful events, many of which were not included in the original ACE checklist, and their impact on adolescent psychopathology. Moreover, only a few to our knowledge use adolescent self-reporting of their stressful experiences (Brockie et al., 2015; Duke et al., 2010). The advantage of self-reporting of events is a valuable means of accessing information from adolescents directly and addresses some key limitations of the extant literature that use retrospective recollection of adversity in adulthood making recall bias more likely or use caregiver reports of adversity due to the sensitive nature of questioning.

The current study attempted a conceptual expansion of ACE categories to include items from the Stressful Life Events Checklist along with a measure of multiple deprivation. The Stressful Events Checklist was developed following extensive piloting in schools and in an adolescent psychiatric unit established by the international CASE study (see Madge et al., 2008). These stressful events include (see measures section) relationship difficulties, serious illness of a family member/friend, suicide or self-harm of a family member/friend, physical/sexual abuse, worries about sexual orientation, being bullied, academic difficulties, and having trouble with the police (Madge et al., 2011; Santiago et al., 2011). These events may deleteriously impact on adolescent development as young people exposed to multiple stressors are more likely to have difficulty forming and maintaining friendships (Borelli & Prinstein, 2006; McMahon et al., 2020; Rudolph et al., 2000), are more at risk of dropping out of school (Wolpow et al., 2009), being unemployed as adults (Kim-Cohen et al., 2003), and experience poverty throughout their lives (Santiago et al., 2011). Moreover, young people who experience relational difficulties or have a family member who have self-harmed or attempted suicide are at an elevated risk of depression, anxiety, and suicidality (Andersen & Teicher, 2008; Bridge et al., 2006; Tidemalm et al., 2011). These young people, many of whom are living in deprived communities, are at a higher risk of experiencing maltreatment, witnessing domestic violence, community violence, and face a much higher chance of being placed on the child protection register, or in out-of-home care (Busso et al., 2017; McCartan et al., 2018). Indeed, stressful life events such as loss, deprivation, injury, and perceived threat are causal factors in the development of major depressive disorder and generalized anxiety (Nishikawa et al., 2018; Spinhoven et al., 2010).

### Aims and Objectives

The aims of the present study were to attempt a conceptual expansion of the ACEs gamut experienced by young people to include other stressful events that may be precursors to adolescent psychopathology (1) to examine the prevalence of self-reported stressful events and associated psychopathology within an adolescent sample in Northern Ireland, (2) to utilise latent class analysis to assess associations between stressful event profiles and subsequent psychopathologic responses, (3) to determine the role of socio-economic area deprivation and its impact on the relationship between adolescent stress profiles and adolescent psychopathology.

#### ***Hypotheses***

1. It is expected that those reporting multiple exposures to stressful events will support a dose–response relationship with adolescent psychopathology.
2. Possible latent class profiles may indicate a low-adversity class and a high-adversity class, with adolescents in the high adversity class being more at risk of psychopathology.
3. Finally, it is expected that those in a high-adversity class will be comprised of adolescents from the most socio-economic deprived areas.

## Design and Participant Sample

Secondary analysis of a cross-sectional survey conducted with a total of 864 (56% female, n = 480) 11–18-year old’s who consented to participate in a school-based survey in four post-primary schools (two secondary schools, one boys grammar, and one girls grammar school) in the North-West region of NI (REC reference: /12/0322). All pupils aged 11 to 18 years (*M* = 13.65; *SD* = 1.76) were invited to participate (n = 4594; 11–14 years n = 583; 15–18 years n = 281). Data for the study were drawn from a modified version of the CASE Study questionnaire, a more detailed methodology is described elsewhere (Madge et al., 2008).

## Measures

### Stressful Life Events

The life events were developed following extensive piloting in schools and in an adolescent psychiatric unit (see Madge et al., 2008). This questionnaire included 20 questions relating to stressful life events experienced in the past 12 months and/or more than a year ago. For the purpose of analysis these variables were collapsed into dichotomised yes, no, responses. Sample items included, *have you had difficulty in making or keeping friends? have you been bullied at school? have your parents separated or divorced? have your parents any serious arguments or fights? have you been seriously physically abused? has anyone among your family or friends completed suicide? has anyone among your family attempted suicide or deliberately self-harmed?* has anyone *forced you to engage in sexual activities against your will?*

### Anxiety and Depression

Anxiety and depression symptomology were measured using the Hospital Anxiety and Depression Scale (HADS; Zigmond & Snaith, 1983). This questionnaire includes two 7-item subscales for anxiety and depression using Likert scaled items. Items for each sub-scale are summed and ranges from 0–21, with higher scores indicating higher levels of anxiety and depression. The scale is considered to have excellent psychometric properties. Within this study the internal consistency coefficient tested using Cronbach’s alpha indicated α = 82 for anxiety and α = 66 for depression.

### Internalising and Externalising Behaviours

The proportion of adolescents in schools reporting internalising and externalising behaviours were measured using the child self-report Strengths and Difficulties Questionnaire (SDQ; Goodman, & Goodman, 2009). The SDQ is a 25-item scale comprising five sub-scales. Four sub-scales represented problem behaviours (Emotional Symptoms, Peer Problems, Hyperactivity, and Conduct Problems) and one sub-scale represented Pro-Social Behaviour. Total difficulties (Broad Psychopathology; α = 82) was calculated by adding the scores for externalising behaviours (i.e., Hyperactivity and Conduct Problems; α = 75) and internalising behaviours (i.e., Emotional Symptoms and Peer Problems; α = 78), with higher scores on each scale indicating higher levels of difficulties experienced.

### Area Stress

Area stress was calculated by collapsing individual postcodes into small neighbourhood deprivation scores. In NI these neighborhoods are called Super Output Areas (SOAs) with populations around 2000. There are 890 SOAs in NI, with each area designated with an index of multiple deprivation score. Scores are ranked by order, with the most deprived areas (rank 1) to the least deprived areas (rank 890). This study used the index of multiple deprivation rank score as a proxy for family, socio-economic circumstances (NISRA, 2019).

## Data Analysis

The binary coded stressful life events items (n = 20) were analysed using latent class analysis (LCA). LCA is a statistical method that is used to categorize underlying homogenous classes or groups from categorical multivariate data (Shevlin & Elklit, 2008). LCA reflects not only the number of stressful events endorsed, but also outlines the overall endorsement pattern (Xian et al., 2008). It is a person-centered approach that classifies unobserved subpopulations into latent classes depending on observed item endorsement (Wang & Wang, 2019). Methodology details applied within this study are available elsewhere (Asparouhov & Muthén, 2014; Bakk & Vermunt, 2016; Nylund-Gibson et al., 2019). Both conceptual consideration and statistical fit indices of the latent class profiles guided decisions concerning the most suitable class model (McBride et al., 2010). Class profiles were estimated beginning with a one-class model, with additional classes sequentially added until fit indices deteriorated. The fit indices included the Akaike Information Criterion (AIC; Akaike, 1987), the Bayesian Information Criterion (BIC; Schwartz, 1978), the sample-size-adjusted BIC (SSABIC; Sclove, 1987), the Lo-Mendel-Rubin Likelihood Ratio-Test (LMR_LRT; Lo et al., 2001), and entropy (Ramaswamy et al., 1993). Lower values on the AIC, BIC, and SSABIC suggest good model fit (Lanza et al., 2007). The model comprising the lowest BIC values indicates the most reliable and best fitting model among the measured set of classes (Nylund et al., 2007). The LRT compares models that comprise different number of classes. If the LRT value is non-significant preference for the model with one less class is advised as a better explanation of the data (Wang & Wang, 2019). Entropy value, which ranges from 0 to 1, is a measure of the classification accuracy regarding respondents’ class placement based on their model-based posterior probabilities (McBride et al., 2010). Higher entropy values indicate more accurate classification of latent class membership (Ramaswamy et al., 1993).

Following identification of the best fitting class profile model and in order to verify the validity of the classes, socio-demographic covariates of gender, age, and SOA scores were added to the model to identify which socio-demographic factors were significantly related to membership of a given class. To assess whether class profiles differed in relation to adolescent psychopathologic responses (SDQ; internalising/externalising behaviours, Conduct Problems, Peer Problems, Hyperactivity, Emotional Symptoms, Pro-social Behaviour, depression, and anxiety), means for these outcome variables were elicited and compared across class profiles utilising the BCH method (Bakk & Vermunt, 2016; Nylund-Gibson, et al., 2019). This approach restricts shifts in latent classes associated with the predominant three-step approach and is preferable to one-step analysis in that the development of class profiles is not confounded by an observed covariate or distal outcome (Nylund-Gibson et al., 2019).

The above analysis was conducted in Mplus version 8.2 (Muthén & Muthén, 1998a, b—2018). The default estimator was robust maximum likelihood (MLR). To avoid a local maxima solution, 500 random starting values were used in the initial stage with 10 optimisations in the final stage of convergence. Considering possible nesting effects, a dummy variable of SOA was included as a clustering variable in the analysis adjusting the standard errors of the estimates (Holt et al., 2017). Logistic regression was used to assess associations between class membership, gender, age, and deprivation scores. The odds ratios indicated the expected likelihood of endorsing a given variable compared with a reference group (Shevlin & Elklit, 2008). Regression analysis was used to investigate whether class membership predicted psychopathologic response.

## Results

The sample consisted of 864 students (see Table 1). The mean age of the students M = 13.65 SD 1.76, minimum = 11 and maximum = 18. Females accounted for 55.6% of the sample. The sample consisted of 96.3% Caucasian, with most students living with both their parents (71.2%). Within the overall sample, 89.6% reported 1 or more stressful event, 46.1% reported 4 or more, with 9.8% reporting 9 or more (M = 3.94, SD = 3.21).

**Table 1:** Sample Demographics

Sample (n = 864)		%	M (SD)
	Age		13.65 (1.76)
	11 years	8.1	
	12 years	24.9	
	13 years	16.9	
	14 years	17.6	
	15 years	20.1	
	16 years	4.3	
	17 years	5.1	
	18 years	3	
Gender			
	Female	55.6	
Ethnicity			
	Caucasian	96.3	
Living arrangements			
	Lived with both parents	71.2	
	Lived with one parent	20.1	
	Lived with one parent and stepparent	6.3	
	Lived with another family member	1	
	Lived with other	1.4	
Stressful event score			3.94 (3.21)
	1 or more	89.6	
	4 or more	46.1	
	9 or more	9.8	

For the initial analysis, multiple independent sample t-tests were conducted to determine whether gender differences in individual characteristic measures of stressful events and outcome variables were observed within the student sample. Results indicate female students experiencing significantly more internalising problems t(862) = -4.49 *p* < 0.001, Emotional Symptoms t(859.80) = -8.087 *p* < 0.001, Pro-social Behaviour t(725.05) = -8.724 *p* < 0.001, and anxiety t(861.34) = -4.22 *p* < 0.001 than their male counterparts, with male students experiencing significantly more externalising problems t(862) = 2.390 *p* = 0.017, Conduct Problems t(784.01) = 4.704 *p* < 0.001, and depression t(862) = 3.26 *p* < 0.001.

### Latent Class Analysis (LCA)

A series of LCA models were estimated beginning with one through to five classes (see Table 2 for fit statistics). Fit indices suggested that the three-class solution was optimal. The BIC was lowest for the three-class solution, whereas the AIC and the SSABIC were lowest for the five-class solution. However, the LMR was non-significant in the four and five-class solutions, suggesting that the three-class model should be accepted. The three-class solution also produced the highest entropy value (0.856).

**Table 2:** Fit statistics for the unconditional LCAs for 1–5 classes

Model	Loglikelihood	AIC	BIC	SSABIC	Entropy	LMR (*p*)
1	-7302.304	14,644.609	14,739.467	14,675.953	-	
2	-6619.891	13,321.783	13,516.241	13,386.037	.814	1355.225 (.000)
**3**	**-6510.489**	**13,144.978**	**13,439.036**	**13,242.143**	**.856**	**217.271 (.0101)**
4	-6443.459	13,052.918	13,446.577	13,182.994	.804	133.120 (.8152)
5	-6388.794	12,985.588	13,478.847	13,148.575	.767	108.563 (.281)

Bold print indicates the best fit statistic across the five models

*AIC* Akaike Information Criterion, *BIC* Bayesian Information Criterion, *SSABIC* sample-size adjusted BIC, *LMR-LRT* Lo–Mendel–Rubin Likelihood Ratio Test

**Fig. 1:**
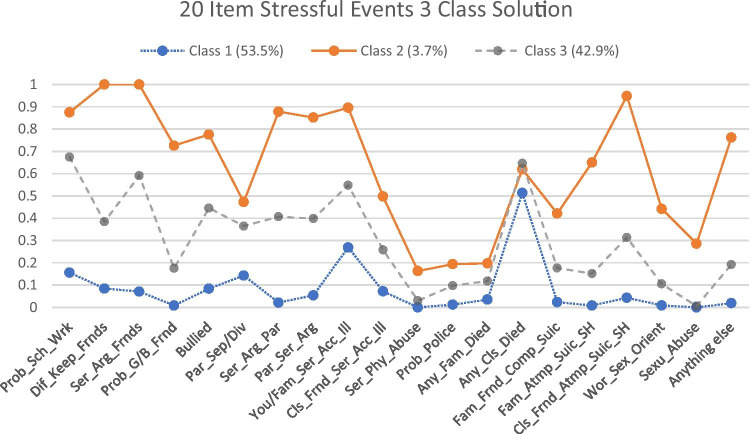
Latent class profiles for 3-class solution showing probabilities of item endorsement

The latent class profile plot (see Fig. 1) displays the probability that adolescents in each class endorsed a particular stressful event item and presents a visual representation of the degree of separation between classes.

Class 1 comprised the largest class (53.5%) and was characterised by adolescents displaying relatively low probabilities of experiencing each of the 20 stressful events with exception of the item “Has anyone close to you died?”. This class was labelled low-risk (see Table 3). Students in class 2 (3.7%) all endorsed having difficulties making and keeping friends and having serious arguments or fights with friends with estimated probabilities of 1, respectively. They also recorded a high probability of witnessing serious arguments or fights between parents (0.85) and having “serious arguments with either one or both parents” (0.88) along with a very high probability of having close friends that have attempted suicide or deliberately self-harmed (0.95) (DSH).

**Table 3:** Actual and model-estimated response probabilities and odds ratios of item endorsement for the three-class-class model

	Actual N (%) Endorsed	Estimated Response Probabilities			OR of Item Endorsement for Class vs. Class		
		Class 1	Class 2	Class 3	2 vs. 1	2 vs. 3	3 vs. 1
Sample (n = 864)							
N (% of sample)		453 (53.5%)	31 (3.7%)	364 (42.9%)			
Female	480 (55.6%)	54%	82%	53%			
Age mean (SE)	13.66 (.06)	13.20 (.10)	14.94 (.29)	14.12 (.10)			
Deprivation mean (SE)	298 (8.22)	293 (.37)	350 (.58)	300 (.37)			
AvePP		0.938	0.944	0.936			
Stress score mean (SD)	3.94 (3.21)	1.28	13.29	6.46			
Problems w/schoolwork	343 (40.4%)	0.16	**0.88**	0.67	38.46	3.38	11.24
Difficulty keeping friends	209 (24.6%)	0.08	**1.00**	0.38	-	-	6.76
Arguments/Fights w/friends	278 (32.8%)	0.07	**1.00**	0.59	-	-	18.87
Problems w/girl/boyfriend	90 (10,6%)	0.01	**0.73**	0.18	333.33	12.47	25.64
Bullied at school	224 (26.4%)	0.08	**0.78**	0.45	38.46	4.30	8.77
Parents separated/divorced	212 (25%)	0.14	0.47	0.37	5.38	1.56	3.45
Arguments/Fights w/parents	185 (21.8%)	0.02	**0.88**	0.41	333.33	10.50	31.25
Witness parents argue/fight	196 (23.1%)	0.05	**0.85**	0.40	100.00	8.67	11.63
Immediate family’ illness/accident	349 (41.2%)	0.27	**0.90**	0.55	23.26	7.11	3.29
Close friends’ illness/accident	142 (16.7%)	0.07	0.50	0.26	12.82	2.85	4.46
Serious physical abuse	16 (1.9%)	0.00	0.16	0.03	-	6.29	-
Trouble w/police	47 (5.5%)	0.01	0.19	0.10	19.23	2.23	8.62
Immediate family died	65 (7.7%)	0.04	0.20	0.12	6.76	1.83	3.68
Anyone else close died	487 (57.4%)	0.51	0.62	0.65	1.55	0.89	1.73
Family/Friends complete suicide	88 (10.4%)	0.02	0.42	0.18	30.30	3.39	8.93
Family/Friends attempt suicide/DSH	79 (9.3%)	0.01	0.65	0.15	250.00	10.40	21.74
Close friends attempt suicide/DSH	163 (19.2%)	0.04	**0.95**	0.31	500.00	40.15	10.20
Sexual orientation worries	56 (6.6%)	0.01	0.44	0.11	90.91	6.73	13.16
Sexual abuse	11 (1.3%)	0.00	0.29	0.01	-	67.72	-
Any other distressing event	102 (12%)	0.02	**0.76**	0.19	166.66	13.48	12.51

Item probabilities > 0.7 bolded to indicate a high degree of class homogeneity. ORs were not estimated since the probability was 1 or 0

*AvePP* average posterior class probability, *SD* standard deviation, *OR* odds ratio, *Class 1* Low-Risk, *Class 2* Immediate-Risk, *Class 3* At-Risk, *DSH* deliberate self-harm

**Table 4:** Summary of significant tests of mean differences on psychopathologic and behavioral variables across classes

	Class Differences	Estimate (b)	S.E	Est./S.E	*p*	95% CI	Cohen’s D
Depression	1 vs. 2	-3.21	1.36	-2.36	.018	-5.89/-0.54	1.34
	3 vs. 2	-2.82	1.41	-2.00	.046	-0.05/-5.59	0.88
Anxiety	1 vs. 2	-4.50	1.12	-4.01	<.001	-6.70/-2.30	2.02
	1 vs. 3	-2.57	0.47	-5.46	<.001	-3.49/-1.65	0.98
Stress	1 vs. 2	-12.04	0.45	-27.00	<.001	-12.91/-11.17	6.14
	1 vs. 3	-5.17	0.16	-31.68	<.001	-5.49/-4.85	2.72
	3 vs. 2	-6.87	0.45	-15.23	<.001	-5.99/-7.75	2.98
Emotional Symptoms	1 vs. 2	-2.40	0.95	-2.52	.012	-4.26/-0.53	1.39
	1 vs. 3	-1.22	0.26	-4.62	<.001	-1.73/-0.70	0.78
Peer Problems	1 vs. 2	-3.71	0.82	-4.54	<.001	-5.31/-2.11	1.21
	1 vs. 3	-0.72	0.19	-3.73	<.001	-1.10/-0.34	0.47
	3 vs. 2	-2.99	0.78	-3.84	<.001	-1.47/-4.52	0.65
Conduct Problems	1 vs. 2	-14.27	1.98	-7.21	<.001	-18.15/-10.39	1.41
	1 vs. 3	-4.70	0.90	-5.24	<.001	-6.45/-2.94	0.68
	3 vs. 2	-9.57	2.17	-4.41	<.001	-5.32/-13.83	0.63
Hyperactivity	1 vs. 2	-9.89	3.36	-2.94	.003	-18.55/-3.30	1.39
	1 vs. 3	-4.58	1.46	-3.13	.002	-8.35/-1.71	0.75
Pro-social	1 vs. 2	10.05	3.25	3.09	.002	1.68/16.43	0.33
	3 vs. 2	8.66	3.33	2.61	.009	17.23/2.15	0.21
Internalising	1 vs. 2	-24.34	5.09	-4.78	<.001	-34.32/-14.37	1.63
	1 vs. 3	-9.71	2.10	-4.64	<.001	-13.82/-5.61	0.82
	3 vs. 2	-14.63	5.31	-2.76	.006	-4.23/-25.03	0.74
Externalising	1 vs. 3	-5.13	2.30	-2.23	.026	-9.64/-0.61	0.80

*Class 1* Low-Risk, *Class 2* Immediate-Risk, *Class 3* At-Risk

Furthermore, students within class 2 had a 78% probability of endorsing experiences of being bullied. with lower probabilities of endorsing the items relating to experiences of forced sexual activity (p = 0.27) and serious physical abuse (p = 0.16). This class was labelled immediate-risk due to the amount of stress experienced and reported difficulties within their relationships. Class 3 (42.9%) was labelled at-risk and was characterised by relatively moderate probabilities of experiencing each of the 20 stress event items (range 0.01 -,67).

Next the association between class membership and concurrent mental health outcomes were examined while controlling for covariates of age, gender, and SOA (see Table 4). First, the assumption that covariates age, gender, and SOA regression coefficients relate identically to outcomes within each class was tested by means of chi-square difference testing using the log-likelihood values and scaling correction factors obtained under the MLR estimator (Bryant & Satorra, 2012). Three models were fitted: a constrained model in which all associations between the covariates and mental health scores were held equal across classes, an unconstrained model in which all associations were free to vary across classes, and a partially constrained model in which some associations were held equal, and some were allowed to vary across classes. Results indicated that the unconstrained model was a better fit than the fully constrained model (Δχ^2^ (48) = 67.91, *p* = 0.031) and the partially constrained model was a better fit than the fully constrained model (Δχ^2^ (24) = 72.57, *p* < 0.001). Given also that the partially constrained model showed no significant deterioration in fit when compared to the unconstrained model (Δχ^2^ (24) = 17.63, *p* = 0.821), the more parsimonious partially constrained model was retained for analysis.

## Differences in Psychopathologic and Behavioural Responses Across Classes

Respondents in the immediate-risk class reported higher depression scores than those in both the low-risk class (*b* = -3.21, *p* = 0.018, d = 1.34) and the at-risk class (*b* = -2.82, *p* = 0.046, *d* = 0.88). For anxiety scores, both the immediate-risk class and the at-risk class recorded higher levels of anxiety than the low-risk class (*b* = -4.50, *d* = 2.02 and *b* = -2.57, *d* = 0.98, *p* < 0.001 respectively). A similar pattern of results emerged in terms of self-reported stress with higher scores recorded in both the immediate-risk class (*b* = -12.04, *p* < 0.001, *d* = 6.14) and the at-risk class (*b* = -5.17, *p* < 0.001, *d* = 2.72) compared against the low-risk class. The immediate-risk class also exhibited higher average stress scores than the at-risk class (*b* = -6.87, *p* < 0.001, *d* = 2.98).

Emotional problems also tended to be higher among those in both the immediate-risk and at-risk classes compared to the low-risk class (*b* = -2.40, *p* = 0.012, *d* = 1.39 and *b* = -1.22, *p* < 0.001, *d* = 0.78 respectively). Peer problem scores were likewise higher in the immediate-risk class compared to both the low-risk group (*b* = -3.71, *p* < 0.001, *d* = 1.21) and the at-risk class (*b* = -2.99, *p* < 0.001, *d* = 0.65), with higher average scores also evident in the at-risk class compared to those in low-risk (*b* = -0.72, *p* < 0.001, *d* = 0.47). A similar pattern of class differences was evident for conduct problems, hyperactivity, internalizing problems and externalizing problems. Those in the immediate-risk class scored higher average scores on conduct problems than those in both the low-risk group (b = -14.27, *p* < 0.001, *d* = 1.41) and the at-risk group (*b* = -9.57, *p* < 0.001, *d* = 0.63). Similarly, the at-risk class recorded higher scores than the low-risk class (*b* = -4.70, *p* < 0.001, *d* = 0.68). Hyperactivity scores also followed this same general trend with higher scores in the immediate-risk and at-risk classes compared to low-risk (*b* = -9.89, *d* = 1.39 and *b* = -0.4.58, *d* = 0.75 respectively, *p* < 0.01). Pro-social behaviour scores tended to be lower in the immediate-risk class when compared to both low-risk (b = 10.05, *p* = 0.002, d = 0.33) and at-risk (*b* = -8.66, *p* = 0.009, d = 0.21). Finally, internalising problems scores also tended to be higher in the immediate-risk class compared to both the low-risk group (*b* = -24.34, *p* < 0.001, *d* = 1.63) and the at-risk group (*b* = -14.63, *p* = 0.006, *d* = 0.74) with the at-risk class exhibiting higher scores than the low-risk group (*b* = -9.71, *p* < 0.001, *d* = 0.82). Finally, externalising problems scores were higher in the at-risk class compared to the low-risk class (*b* = -5.13, *p* = 0.026, *d* = 0.8).

### Demographic Differences Within Classes

#### Low-Risk Class

Within latent class one (low-risk class) both being female (*b* = 0.73, *p* = 0.003) and being older (*b* = 0.12, *p* = 0.014) were associated with higher emotional problems scores, whilst being male (*b* = -0.55, *p* = 0.003) and living in a more deprived area (b = -0.05, *p* = 0.028) were linked to higher scores on peer problems. In addition, males within this class scored higher on both conduct problems (*b* = -0.72, *p* < 0.001) and hyperactivity (*b* = -0.57, *p* = 0.020) and females recorded higher pro-social behaviour scores (*b* = 1.65, *p* < 0.001). Older adolescents reported higher anxiety (*b* = 0.19, *p* = 0.028) and males scored higher on depression (*b* = -1.73, *p* < 0.001). Furthermore, males scored higher on internalising problems within class one (*b* = -1.30, p < 0.001) and older adolescents reported higher externalising problems (*b* = 0.27, *p* = 0.013).

#### Immediate-Risk Class

Within latent class two (immediate-risk class), females (*b* = 1.94, *p* = 0.050) and older adolescents (*b* = 0.12, *p* = 0.014) exhibited higher emotional problems. Greater area deprivation was associated with higher peer problems (*b* = -0.05, *p* = 0.028) and younger respondents scored higher on conduct problems (*b* = -0.66, *p* < 0.001) and hyperactivity (*b* = -0.43, *p* = 0.022). Furthermore, being female (*b* = 1.28, *p* = 0.035; *b* = 4.42, *p* = 0.006) and being older (*b* = 0.52, *p* = 0.011; (*b* = -0.19, *p* = 0.028) was associated with higher pro-social behaviour and anxiety respectively. Finally, within this class, higher internalizing scores were recorded from younger respondents (b = -1.07, *p* < 0.001) and those from more deprived areas (*b* = -0.46, *p* = 0.003). 

#### At-Risk Class

Hyperactivity scores were also higher among females within this at-risk class (*b* = 0.73, *p* = 0.002) along with higher anxiety (*b* = 2.05, p < 0.001), pro-social behaviour (*b* = 0.58, *p* = 0.028), emotional problems (*b* = 1.94, *p* < 0.001) and externalising problems (*b* = 2.12, *p* < 0.001), and Older adolescents (*b* = 0.12, *p* = 0.014) scored higher on emotional problems. Deprivation was linked to higher scores on peer problems (*b* = -0.05, *p* = 0.028) and younger adolescents reporting higher conduct problems (*b* = -0.21, *p* < 0.001) and hyperactivity (*b* = 0.19, *p* = 0.002). while being older predicted anxiety (*b* = 0.118, *p* = 0.028). Again, being younger predicted internalising problems (*b* = -0.420, *p* < 0.001) and being female predicted externalising (*b*=2.20, *p* <0.001).

#### Logistic Regression

Older students were more likely to be in the immediate-risk class compared to both the low-risk class (OR = 1.7, *p* < 0.001, 95% CI = 1.426—2.036) and the at-risk class (OR = 1.38, *p* < 0.001; 95% CI = 1.233—1.543). Deprivation was not linked to likelihood of class membership.

## Discussion

The present study attempted a conceptual expansion of the ACEs checklist by examining 20 items of the Stressful Life Events Checklist (Madge et al., 2008) within a sample of post-primary school adolescents in N.I. Findings revealed that those most at risk of experiencing adolescent psychopathology had a high probability of encountering relationship issues, experiencing family dysfunction, and/or having a family member undergo a serious illness, and having close friends who deliberately self-harm or have attempted suicide. These findings indicate that ACEs may present in many forms, such as loss, inter-personal relationships, family dysfunction, illness, or having close friends experiencing psychological difficulties. Consequently, this study demonstrates that the original ten ACE categories may be too narrow in focus, and therefore may not encapsulate the full spectrum of ACES. Broadening the scope of the ACE checklist to include other stressful events is recommended as these may also be antecedents to psychopathologic responses.

The study examined the prevalence of self-reported stressful events within the sample. The most common stressful events reported included, having someone close dying, having had or someone in the family having had a serious illness or accident, having had serious arguments or fights with friends and with either or both parents, having difficulty making or keeping friends, being bullied at school, having parents who are separated or divorced, and having close friends or family members attempting suicide or self-harming. Concerningly, over 10% of the sample endorsed having either a family member or friend who completed suicide. Respondents within the sample reported low levels of physical abuse and sexual abuse compared with previous studies (Madge et al., 2011). Nevertheless, the study has shown high reported incidence rates of stressful life events among adolescents in N.I. On average, adolescents indicated that they had experienced approximately four stressful events from the 20-item checklist, with over 40% experiencing seven or more, and only 10% experiencing no stressful events. As expected, participants reporting multiple exposures to stressful events supported a dose–response relationship with adolescent psychopathology, with multiple events significantly predicting more psychopathologic responses. Similar to findings from a systematic review and meta-analysis (Petruccelli et al., 2019), this study revealed adolescent females reporting more stressful events than males, females were also more likely to experience internalising problems, Emotional Symptoms, anxiety, and be more pro-social than their male counterparts. Males were more likely to experience externalising problems, and depression. The graded relationships indicated by the gradual increase in psychopathological responses demonstrated the cumulative effect of stressful life events. The risks for mental ill-health increase significantly according to the number of stressful life events reported. Indeed, studies reporting an adversity score add to the volume of mounting evidence on the relationship between childhood adversities and psychopathology (Chapman et al., 2004; Dube et al., 2001).

Previous studies revealed that childhood adversities do not happen in isolation with co-occurrence common (Armour et al., 2014; Creamer et al., 2001). Using a person-centred approach, latent class analysis of the 20-item checklist responses did not show any distinct typology profiles of stressful event experience (e.g., a sexual abuse class or a peer problems class), rather a continuum from low to high stressful event experience was revealed. These findings demonstrate that within the post-primary schools sampled there seems to be a generally healthy class of students (low-risk class), an unhealthier at-risk class, and an immediate-risk high stress class. The more stressful events experienced, the more internalising and externalising problems these adolescents reported. There was no distinct sexual abuse class, however, consistent with previous research associating sexual abuse with a high risk of poly-victimisation (Barnes et al., 2009), sexual abuse was more commonly reported by respondents of the immediate-risk class. Previous research found that exposure to child sexual abuse was associated with increased risks of psychopathology, including depression, anxiety, Conduct Problems, and suicidal ideation (Fergusson et al., 2008).

These latent classes closely correlate with Shemmings and Shemmings (2011) who reported that approximately 60% of children develop stable, healthy emotional bonds with their parents, whilst 40% do not. These young people are less able to cope with stress or adversity and are more prone to internalizing and externalizing behaviours. In addition, adolescents raised in an environment where family dysfunction is common will often find it difficult to form and maintain healthy relationships. This can lead to lasting psychological problems such as increased anxiety, depression, and suicidality (Cook et al., 2017; Petruccelli et al., 2019). Next, the associations between stressful event classes and subsequent psychopathologic responses were assessed. In accordance with previous findings, adolescents reporting multiple exposures to stressful events supported a dose–response relationship with adolescent psychopathology (Lew & Xian, 2019; McLafferty et al., 2015), with both the at-risk and immediate-risk classes reporting higher anxiety, depression, and worse broad psychopathology than the low-risk class. This study also assessed the role of area deprivation and its impact on the relationship between stressful event classes and adolescent psychopathology. Results indicated that high deprivation did not predict membership of any of the classes, however, the measure did predict Peer Problems within each class individually alongside predicting internalising behaviours within the immediate-risk class. Surprisingly, indicators of deprivation were lowest within the immediate-risk class compared to the other two classes. As the schools involved in this study belong to a region of N.I with historically high deprivation, this anomaly may be explained by a relative lack of variance in deprivation scores within this sample.

Our overall findings reveal that exposure to stressful events are associated with numerous psychopathologic outcomes among adolescents in a dose–response pattern. As adolescents get older, the odds of experiencing mental ill-health increase especially for adolescents who have experienced multiple stressful events. As Burke and Minton (2019) suggest, this finding may be at least partially attributable to academic pressures of examinations in older pupils (Burke & Minton, 2019). Future research should also examine and cross-correlate other potential measures potentially influencing adolescent well-being such as puberty, social relationships, and transitions from junior to senior years.

As our findings illustrate, all young people in the immediate-risk class who have endured multiple stressful events have also reported experiencing relational difficulties. Previous research suggests that adolescents who experience difficulties in school and within their social lives are more likely to encounter feelings of rejection and failure leading them to being more susceptible to Emotional Symptoms including depression (Powell et al., 2020). As adolescents separate from their parents, they depend on their peers more for social support to guide them through this important transition of development. The importance of good friendships, feeling accepted by others, and playing or interacting with others may be important factors ameliorating the onset of depressive symptoms and building healthy self-concepts. Prior findings have shown that self-concept is key to psychological well-being but may be affected by poor social relationships or stressful events (McMahon et al., 2020). Poor quality relationships may impact on health and frequently result in diminished levels of psychological well-being (McMahon et al., 2020), and increased depressive symptomology (Andersen & Teicher, 2008). In turn, depression may generate avoidance and conflictual interpersonal behaviours leading individuals to withdraw from social engagement eliciting further feelings of rejection and deterioration in their social lives and the exacerbation of additional stress (Rudolph et al., 2000).

As for the domains of the SDQ, a straightforward description of how these problems may interact leading to adolescent psychopathology may be that difficulties in each domain have a reciprocal relationship with each other due to one problematic domain precipitating difficulties in other domains (e.g. adolescents with emotional problems are less able to form and maintain healthy peer relationships), or, due to the variables been linked bi-directionally (e.g. each problem domain aggravating the other over a period of time: adolescents with peer problems advance further emotional difficulties and vice versa), or, because of shared etiological factors which impact development of both peer and emotional problems (Mok et al., 2014). As this study demonstrates, other vulnerable factors known to influence emotional problems in adolescence are gender. Females are considered more relationally oriented and exhibit greater affiliative needs especially in adolescence, they are more reactive to peer stress and are more likely to experience internalizing problems in comparison to males (Hankin et al., 2015).

### Limitations

Our overall findings should be considered in light of several limitations. First, the current study was cross-sectional, it was not possible to determine the causal order of stressful events and domains listed in the SDQ. Second, the frequency of individual events, the emotional intensity felt by respondents to these events, and whether these events were daily, weekly, or monthly and so on were not elicited within this study. Any further research may include prospective, longitudinal, and qualitive studies that measure the age of exposure, frequency, and chronicity of exposure, providing a deeper understanding of the implications of stressful events on adolescent psychopathology. Third, the study assumed that each item on the Stressful Life Event Checklist had the potential to contribute equally to adolescent psychopathology. Assessing the effects of each individual stressful event with the potential to identify whether one event in comparison to another had a possible greater impact on adolescent psychopathology was beyond the scope of this paper. Future research may demonstrate the individual impact of each item of the Stressful Life Events Checklist. Fourth, even though the self-rated version of the SDQ was shown to be a reliable and valid method for the assessment of behavioural problems in adolescents (Goodman & Goodman, 2009), caution may be warranted in determining causation not least because such reports are subjective and unverifiable. Better use of triangulation such as adolescent self-report, parental report, along with teacher report would provide a more accurate appraisal of psychopathology in adolescence. Finally, the small size of the immediate-risk class can cause less reliable estimates of class-specific parameters and may diminish substantive meaning of the latent class (Brown et al., 2019).

### Clinical Implications

Our findings reveal that there may be small yet meaningful numbers of adolescents in schools who need immediate intervention to change their life-course. These young people may benefit from individual and family therapies (Das et al., 2016), and programmes aimed at stress reduction (Hofmann et al., 2010). However, the young people who pose as at-risk may often go undetected if they are not disruptive to school life. This cohort may therefore be offered little in terms of interventions or prevention programmes aimed at reducing anxiety, depression, and behavioural problems. Therefore, and in line with the findings of this study, it is suggested that broad intervention or prevention programmes targeting whole-school student mental health, ensuring that every young person learns healthy coping skills in the face of adversity are implemented (Essau et al., 2012). In addition, schools need to ensure that enhancing relational connectedness are at the core of these programmes. Moreover, it is recommended that within post-primary schools all staff be trained to become more ACE-informed and trauma responsive. An ACE-informed and trauma-responsive whole-school approach requires school staff to support all students regardless of exposure to risk. Training would provide staff the knowledge and skills to act as an always available adult (Bellis et al., 2017), empowering students to seek support when needed, and enabling staff to build resilience in students to protect against deleterious outcomes associated with ACEs (Barton et al., 2018).

Additionally, the value of this research demonstrates the significance of early screening for ACEs in adolescent development within both clinical and child protection services along with other settings that serve as a significant point of entry to services for adolescents such as schools and primary care. However, limitations to screening that employ available instruments to survey ACEs are numerous. For example, it is important to note the paucity of clinical guidelines available defining poly-victimisation and how such information is combined in assessing risk. Currently, there are no available existing measures that provide an exhaustive list of possible ACEs. In addition, ACEs are weighted equally on existing instruments, it seems improbable that these individual ACE items confer equal risk of stress related traumatization and their long-time effects. Consequently, results attained through existing measures can only provide a rough estimate of the level of ACEs experienced by adolescents. Furthermore, quantifying the total number of ACEs without enquiring about protective factors may lead to decisions pertaining to clinical care and services based upon misclassification of risk (Anda et al., 2020). Further research may test prediction models that account for risk, including poly-victimisation, abuse, loss, peer relationships, household dysfunction, and indices of deprivation, as well as protective factors such as resilience (Fergusson et al., 2008). Models accounting for both risk and protective factors may inform the development of more sophisticated assessment measures with the potential to target predominantly high-risk adolescents and improve the allocation of scarce and diminishing resources. Thus, clinical interventions need to have the ability to span the potential range of young people’s difficulties, identify individual resilience that can be bolstered through therapy along with the need for both whole family and whole school approaches to intervention.

## Conclusion

This study attempted a conceptual expansion of the ACE checklist utilizing the stressful life events and problems checklist (see Madge et al., 2008) in adolescents aged between 11 to 18 years. To our knowledge, no such study has been undertaken in this way before with this age group. Our findings demonstrated that those at most risk of adolescent psychopathology had the highest probability of encountering interpersonal relationship issues, experiencing family dysfunction and illness, loss, and having close friends experiencing psychological difficulties. Consequently, this study demonstrated that the original ten ACE categories may not capture the wide range and complexity of childhood adversity and supports the inclusion of peer relationships/difficulties along with indicators of deprivation to the ACE checklist as these were found to be strong predictors of psychopathology. Further, the number of stressful events reported within the sample had a graded relationship to broad and specific psychopathology in adolescence. These findings support existing literature on the association between the cumulative impact of childhood adversity and adolescent psychopathology. Latent class analysis revealed a three-class solution consisting of a low-risk, at-risk, and immediate-risk of adolescent psychopathology groupings. Rather than only focusing resources aimed at ameliorating psychopathology at the immediate risk group, this study also supports broad intervention or prevention programmes targeting whole-school mental health. Finally, living in an economically disadvantaged area has many social and emotional implications for adolescent development, including internalizing problems and Peer Problems. Efforts to improve adolescent mental health outcomes should spotlight socioeconomic inequalities along with early identification, and the implementation of prevention, and intervention strategies.

## Data Availability

On request.
